# Giant internal carotid artery aneurysms and porcelain aorta in an elderly patient with Marfan syndrome

**DOI:** 10.1002/ccr3.6190

**Published:** 2022-08-09

**Authors:** Hiroki Yagi, Norifumi Takeda, Yumiko Hosoya, Haruo Yamauchi, Issei Komuro

**Affiliations:** ^1^ Department of Cardiovascular Medicine The University of Tokyo Hospital Tokyo Japan; ^2^ Marfan Syndrome Center The University of Tokyo Hospital Tokyo Japan; ^3^ Department of Therapeutic Strategy for Heart Failure The University of Tokyo Hospital Tokyo Japan; ^4^ Department of Cardiac Surgery The University of Tokyo Hospital Tokyo Japan

**Keywords:** elderly patient, internal carotid artery aneurysm, Marfan syndrome, porcelain aorta

## Abstract

Marfan syndrome (MFS) an inherited disorder caused by *FBN1* gene variants, is well known to cause lethal aortic aneurysm and dissections at a relatively young age. Here, we report giant internal carotid artery aneurysms (ICAAs) and porcelain aorta in an elderly patient with MFS.

## Graphical abstract

Recent medical and surgical management has improved the long‐term prognosis of MFS and the number of elderly patients increases. Unfamiliar late arterial complications might be kept in mind including ICAAs, because MFS arteries are considered to be easily affected by lifestyle‐related diseases with age.

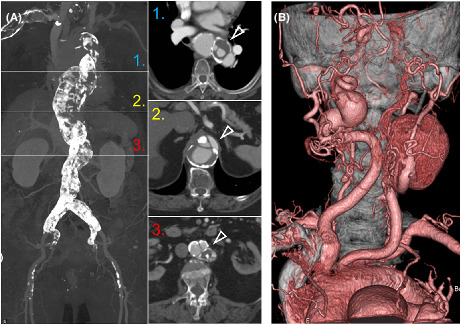

The patient is a 79‐year‐old obese woman with a BMI of 31.6 and complicated with hypertension, dyslipidemia, and diabetes mellitus. At age 61, a CT scan identified chronic thoracoabdominal aortic dissection (TAAD) involving multiple visceral and cervical arteries and with severe calcification (porcelain aorta) (Figure 1A). At age 76, asymptomatic and marked enlarged bilateral internal carotid artery aneurysms (ICAAs) with tortuosity were incidentally depicted (Figure 1B), and conservative management was chosen because of her greater risk of surgical treatment. In April 2017, she was referred to our hospital and was diagnosed with Marfan syndrome (MFS) based on the presence of ectopia lentis and TAAD and a previously reported *FBN1* pathogenic variant (c.1709G>C; p.Cys570Ser); however, she died suddenly due to an unknown cause a few months later. MFS is an autosomal dominant connective tissue disorder with a genetic predisposition to aortic aneurysms and dissections at a relatively young age.^1^ Recent advances in the medical and surgical management have improved life expectancy. ICAAs and porcelain aorta are rarely complicated in MFS;^2^ however, as the number of elderly patients increases, these unfamiliar late arterial complications might be kept in mind to develop more comprehensive management, because MFS arteries are considered to be easily influenced by lifestyle‐related diseases.

**FIGURE 1:**
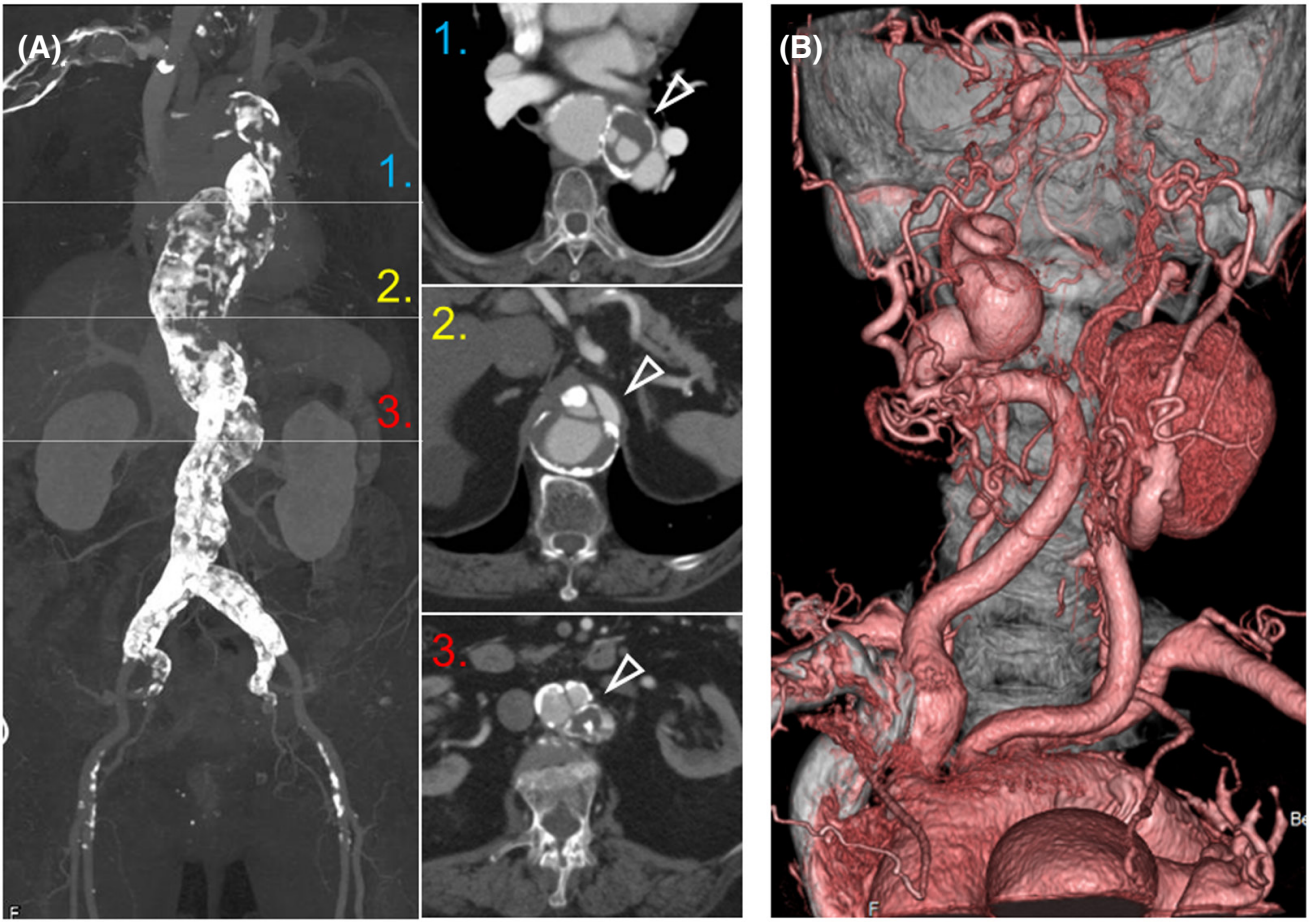
CT findings of the present case. (A) The severely calcified thoracoabdominal aorta image (left panel) with corresponding contrast‐enhanced CT images (right panels). Arrowheads indicate aortic dissections. (B) Three‐dimensional CT angiography. The right and left internal carotid arterial aneurysms were 22 and 56 mm in maximum diameter, respectively.

## AUTHOR CONTRIBUTIONS

Hiroki Yagi (HY), Norifumi Takeda (NT), Yumiko Hosoya (YH), and Haruo Yamauchi (HY) were directly involved in the management of the case. Issei Komuro revised the manuscript critically for important intellectual content. All authors approved the content of the manuscript and confirmed the accuracy or integrity of any part of the work.

## FUNDING INFORMATION

The authors have not declared a specific grant for this research from any funding agency in the public, commercial, or not‐for‐profit sectors.

## CONFLICT OF INTEREST

The authors declare no conflict of interest.

## ETHICAL APPROVAL

The genetic analysis was approved by the University of Tokyo Hospital ethics committee (G‐1538), and this case report was conducted in accordance with the Declaration of Helsinki.

## CONSENT

Written informed consent was obtained from the patient to use the data and pictures and publish this report in accordance with the journal's patient consent policy.

## Data Availability

The data that support the findings of this study are available on request from the corresponding author.
